# Prostaglandin Pathway Polymorphisms and HsPDA Treatment Outcomes in Preterm Infants below 32 Weeks: Pilot Study

**DOI:** 10.3390/genes16111271

**Published:** 2025-10-28

**Authors:** Marcin Minta, Grażyna Kurzawińska, Zuzanna Banach Minta, Agnieszka Seremak Mrozikiewicz, Dawid Szpecht

**Affiliations:** Department of Neonatology, Karol Marcinkowski University of Medical Sciences in Poznan, ul. Polna 33, 60-535 Poznań, Poland; gkurzawinska@ump.edu.pl (G.K.); zuzbanach@gmail.com (Z.B.M.); dawid.szpecht@poczta.fm (D.S.)

**Keywords:** hemodynamically significant ductus arteriosus, patent ductus arteriosus, risk factors, genetics factors, pharmacological ligation, preterm newborns

## Abstract

Background: Despite substantial advances in neonatology, the management of patent ductus arteriosus (PDA) in preterm infants continues to pose a major clinical challenge. The presence of PDA may exacerbate circulatory instability, contribute to tissue hypoxia, and significantly increase the risk of prematurity-related complications. Risk factors for patent ductus arteriosus (PDA), including its hemodynamically significant form (HsPDA), have been consistently associated with lower gestational age, low birth weight, the requirement for invasive mechanical ventilation, and lack of antenatal corticosteroid exposure. With the advent of novel diagnostic approaches, growing attention has been directed toward the genetic determinants of various neonatal conditions. Genetic variability has been extensively documented as a factor influencing drug metabolism, thereby modulating dose-dependent responses and resistance to standard pharmacological interventions. Methods: The study population comprised neonates delivered before 32 completed weeks’ gestation to assess the potential impact of polymorphisms in genes associated with the prostaglandin pathway on the response to pharmacological treatment of HsPDA. Results: Among the identified polymorphisms, one demonstrated a statistically significant association with treatment success. Additional analyses were performed to determine whether therapeutic efficacy differed according to the drug administered. Conclusions: We propose that further research, particularly studies incorporating diverse ethnic populations, may provide valuable insights into the underlying the pathophysiology of PDA and contribute to the development of more effective treatment strategies.

## 1. Introduction

Premature newborns are at risk of developing many complications resulting from immaturity. One of them is the development of a patent ductus arteriosus (PDA), which can lead to organ hypoperfusion, pulmonary congestion, and the development of heart failure, a condition called hemodynamically significant ductus arteriosus (HsPDA) [1]. Closing of the DA is associated with a decrease in the concentration of prostaglandins in the blood, which are responsible for maintaining its patency. This process may be weakened in the case of premature newborns or in the course of an inflammatory process. Key risk factors for PDA include lower gestational age, low birth weight, and the requirement for mechanical ventilation. Additional risk factors, both related to the increased risk of occurrence and lack of response to pharmacological treatment, are believed to be genetic. The basis was studies on monozygotic twins and conducted on animal models [2,3,4,5].

For an extremely premature neonate, blood theft through a patent PDA leading to hypoperfusion is a condition that can quickly lead to a breakdown of the body’s homeostasis. Early diagnostics and prevention are required to provide the opportunity to intervene before complications occur [6].

Ductus arteriosus causing complications resulting from hypoperfusion or pulmonary parenchyma congestion is eligible for pharmacological treatment. The drugs approved for pharmacological ligation in Poland are ibuprofen, and in the case of contraindications to its use, paracetamol [7,8,9,10,11,12,13]. Varying efficacy in the case of pharmacotherapy may result from different enzyme activities affected by specific drugs through different polymorphisms of the genes encoding specific enzymes or receptors.

Although the influence of the genes discussed herein on the development and outcomes of treatment for PDA has not been previously investigated, we identified several studies examining associations between these polymorphisms and other clinical conditions [14,15,16,17,18,19,20,21,22]. We interrogated polymorphisms in enzyme and receptor genes within the arachidonic acid–prostaglandin pathway for associations with pharmacologic treatment response. In particular, we interrogated SNPs variants in phospholipase A2 (rs10798059, rs1549637, rs4375, rs1805017, rs1051931), cyclooxygenase-1 (rs1236913), prostaglandin synthase 2 (rs13283456), and the prostaglandin E2 receptor EP4 (rs4613763).

In reviewing the literature related to the studied genes, we identified a meta-analysis assessing the impact of the PTGS2 polymorphism on aspirin resistance, which included both Asian and European populations. The analysis demonstrated the presence of a polymorphism associated with increased aspirin resistance, and differences in its prevalence were observed between the two populations [5]. Another meta-analysis investigated polymorphisms in the PTGER4 gene in relation to the development of inflammatory bowel disease. Depending on the specific variant, a significant association was identified between the presence of the polymorphism and disease susceptibility [23,24].

These findings suggest that genes involved in prostaglandin synthesis and signaling—key mediators of inflammatory processes—may contribute to the pathogenesis of a variety of clinical conditions.

## 2. Methods

### 2.1. Definitions

Patent ductus arteriosus was defined as echocardiographically confirmed ductal patency—persistent flow between the pulmonary artery and the aorta—beyond postnatal day 5. Hemodynamic significance was classified according to neonatal care standards. (Table 1). Diagnosis of HsPDA required echocardiographic evaluation of ductal diameter, flow in the descending aorta distal to the ductal insertion, and end-diastolic flow in the left pulmonary artery.

### 2.2. Diagnostic Criteria for PDA

Diagnostic assessment was performed by transthoracic echocardiography, the current gold standard for detecting and monitoring ductal patency. Examinations were performed by credentialed personnel with documented training. Hemodynamic significance was adjudicated according to the most recent guidelines of the Polish Neonatal Society. In accordance with current standards, echocardiography was performed on postnatal day 2 and repeated at 24 h intervals until ductal closure. The efficacy of pharmacological treatment was defined as complete ductal closure confirmed by echocardiographic examination. All echocardiograms were acquired on a Samsung (Suwon, Republic of Korea) V8 ultrasound platform equipped with a PA4-12B probe.

### 2.3. Study Design and Data Collection

The study cohort comprised 99 neonates born at 24–32 weeks’ gestation and admitted to the Neonatal Intensive Care Unit of the Ginekologiczno-Położniczy Szpital Kliniczny (GPSK), Poznań, Poland, during 2022–2023.

Patients in whom it was not possible to obtain complete clinical or genotyping data—such as those who died before the completion of hospitalization, or those with congenital heart defects or other major anomalies affecting clinical status—were excluded from the study. To ensure data quality and population consistency, Hardy–Weinberg equilibrium analysis was performed for all investigated SNPs prior to inclusion in the statistical evaluation.

Clinical data were retrospectively abstracted from medical records maintained during the hospitalization. Blood necessary for genetic testing was collected during blood collection for standard tests.

### 2.4. Ethics

Medical data were collected from available patient documentation. Blood necessary for genetic testing was collected during blood collection for standard tests to minimize additional procedures. Care was also taken to minimize the amount needed to perform genotyping (0.5 mL). Parents or legal guardians were informed in advance about the planned procedures and their purpose, and written informed consent was obtained. Confidentiality was protected by restricting involvement to essential study personnel and encrypting all data. Ethics approval was granted by the Bioethics Committee of the Poznan University of Medical Sciences (Resolution No. 96/22). Funding for this work was provided by the National Science Centre, Poland (NCN), grant No. 2021/05/X/NZ5/01430.

### 2.5. Studied SNPs

The analyzed genes encode components of the arachidonic acid cascade culminating in prostaglandin synthesis. Selection of individual polymorphisms was based on analysis of their prevalence in our regional population.

*PTGS1*, located on chromosome 9q32–q33.3, encodes prostaglandin-endoperoxide synthase 1 (cyclooxygenase-1, COX-1), which converts arachidonic acid to prostaglandin H_2_. According to the available literature, the polymorphism within this gene is associated with a reduction in cyclooxygenase-1 activity. The extent of enzymatic activity decrease varies depending on the allelic variant, with a markedly greater reduction observed in the recessive form [25].

*PTGES2*, mapped to chromosome 9q34.11, encodes microsomal prostaglandin E synthase-2, which converts prostaglandin H_2_ to prostaglandin E_2_. The polymorphism may influence pre-mRNA splicing, resulting in the production of alternative protein isoforms. This phenomenon affects both the enzymatic activity and the plasma concentration of the protein. The most robust evidence in this regard originates from in vitro studies. However, researchers emphasize that findings from cohort studies are influenced by additional variables, including sex and hormonal factors, which warrants further investigation [26].

*PTGER4*, mapped to chromosome 5p13.1, encodes the prostaglandin E_2_ receptor EP4, which mediates cellular PGE_2_ signaling. The polymorphism may affect mRNA splicing or the binding of regulatory factors, thereby influencing gene expression and protein function. Increased gene expression associated with polymorphisms such as rs4495224 and rs7720838, which affect receptor encoding, results in an augmented biological response. Available evidence indicates that these variants are linked to an elevated risk of Crohn’s disease and may also contribute to a higher incidence of patent ductus arteriosus [27,28].

This pathway additionally involves multiple phospholipase A_2_ isoform genes.

*PLA2G4A*, mapped to 1q31.1, exhibits genetic variation that may lead to either increased or decreased gene expression depending on the specific polymorphism. The rs12746200 variant is associated with reduced enzymatic activity, whereas rs2049963 corresponds to increased activity, which in turn may contribute to a diminished response to pharmacological treatment [29,30].

On chromosome 19q13.3, *PLA2G4C* encodes a cytosolic PLA_2_ isoform; some SNPs have been reported to increase expression [31]. At present, the available literature does not describe the influence of these polymorphisms on the occurrence of patent ductus arteriosus. However, studies concerning colorectal cancer and schizophrenia have reported that certain polymorphisms may exacerbate disease severity and are associated with a less favorable prognosis in malignancy [32].

*PLA2G6*, mapped to chromosome 22q13.1 (the polymorphism may lead to modulation of mRNA splicing or result in the expression of alternative isoforms). For the SNPs rs132985, rs2284063, and rs2267369, no consistent or statistically significant effect on expression or enzymatic activity has been demonstrated [33].

*PLA2G7*, located on chromosome 6p21.2. The rs1051931 polymorphism results in an amino acid substitution, where valine is replaced by alanine, leading to reduced enzymatic activity. Research is still ongoing on the impact of decreased expression on the development of disease [34]. The rs1805017 polymorphism, which involves an arginine-to-histidine substitution, has been associated with increased enzymatic activity [35].

### 2.6. Testing Methodology

Blood for genotyping was collected into EDTA (ethylenediaminetetraacetic acid)–anticoagulated tubes and stored at −20 °C until DNA extraction. Genomic DNA was isolated from nucleated blood cells using the QIAamp DNA Mini Kit (QIAGEN, Hilden, Germany) according to the manufacturer’s instructions.

The variants listed in Table 2 were genotyped by polymerase chain reaction–restriction fragment length polymorphism (PCR–RFLP).

PCR–RFLP products were resolved on 2.5% agarose (1× TBE) at 120 V for ~2 h with a 50-bp ladder. Following UV transillumination, banding patterns were documented and genotypes were assigned based on the expected restriction fragment sizes. PCR primers were designed using Primer3Plus (v3.3.0). SNP-specific restriction endonucleases were selected with NEBcutter (v3.0; https://nc2.neb.com/NEBcutter2/, accessed on 8 October 2024).

## 3. Statistical Analysis

Comparisons of dichotomous variables were performed using chi-square tests with or without Yates’ continuity correction, as appropriate. Odds ratios (ORs) with 95% confidence intervals (CIs) were estimated. Two-sided *p* values < 0.05 were considered statistically significant.

Power calculations were based on baseline incidences of PDA (33.6%) and HsPDA (12.1%) observed in our hospital during the two study years. In the present cohort (n = 99), the observed prevalences were 36.4% (PDA) and 21.2% (HsPDA). With α = 0.05, the achieved power was 8.9% for PDA and 74.3% for HsPDA. Multiple comparisons were controlled using the Bonferroni method (eight tests), yielding an adjusted significance threshold of α_adj = 0.006 (i.e., P_corr with 0.05/8).

Data were analyzed with GraphPad Prism (version 2024; GraphPad Software, San Diego, CA, USA) and Statistica (version 10, 2011; StatSoft, Inc., Tulsa, OK, USA).

## 4. Results

The study cohort comprised 99 neonates born at 24–32 weeks’ gestation (median, 30 weeks). 45 were female (45/99, 45.5%) and 54 were male (54/99, 54.5%). PDA was diagnosed in 36 infants (36/99, 36.4%), of whom 21 (21/99, 21.2%) had hemodynamically significant PDA (HsPDA).

Analyzing risk factors, it was found that PDA occurs significantly more frequently among newborns with a birth weight of <1000 g. PDA was statistically significantly more likely to require invasive ventilation. In the study group of newborns with PDA, complications of prematurity, such as NEC, BPD, and ROP, were significantly more common. Data are presented in Table 3.

21 (21.11%) patients were treated with ibuprofen (n = 3; 3.03%), paracetamol (n = 14; 14.14%), and both (n = 4; 4.04%). The characteristics of the group are shown in Table 3.

Among the analyzed polymorphisms, we noticed a statistically significant influence of rs4613763 in the codominant variant on the success of the applied pharmacological therapy. We did not find any statistical significance difference in the effectiveness of treatment HsPDA with Paracetamol vs. Ibuprofen. All results are presented in Table 4 and Table 5.

## 5. Discussion

To our knowledge, no prior study has directly interrogated genetic variation across the arachidonic acid–prostaglandin pathway, including both enzymatic and receptor genes and its principal regulatory nodes. The results we have obtained should be an introduction to further analysis of the gene variants we have studied, as well as the search for further genetic conditions that may affect the success of the pharmacological treatment used. Due to the relatively homogeneous ethnic group of newborns included in the study due to the profile of patients hospitalized in our hospital, we believe that expanding the search to other countries may bring more valuable information.

Analyzing the effectiveness of ibuprofen and paracetamol in relation to individual polymorphisms in patients included in our study, we conclude that in the studied group there is a relationship between variants of individual polymorphisms and their influence on the effectiveness of therapy, such as rs4613763 polymorphisms. Therefore, we should base our diagnostic decisions at the moment mainly on the clinical condition of the neonate together with drugs contraindications and knowing that they have similar effectiveness but differ in possible complications.

The clinical response to nonsteroidal anti-inflammatory drugs (NSAIDs) administration to the neonate with respect to PDA closure is well known [10].

There is a noticeable difference in the concentration of drugs dosed on body weight in individual patients resulting from different activity of enzymes responsible for the metabolism of substances. Their activity may be related to the maturity of the child, its clinical condition, but as studies show, also genetic variability between individuals [14,36,37]. There are scientific studies conducted for cytochromes CYP2C8 and CYP2C9 describing the variability of the reaction and clearance of ibuprofen and consequently the concentration of the drug in plasma depending on its polymorphism. For genetic variants associated with reduced clearance, through increased concentration of the substance in the blood we observe a more intensive therapeutic effect [15,16,17].

Similarly to the previously described ibuprofen, also for the paracetamol used in the treatment of ductus arteriosus in newborns, there are scientific reports about variable pharmacokinetics and the achieved concentration of the drug in the blood [18]. Paracetamol is metabolized in the liver by UGT1A1 and UGT1A6 and SULT1A1 and SULT1A3. [19,20]. It has been shown that individual single-gene polymorphisms of the genes encoding these enzymes may affect the metabolism and elimination rate of the substance, and thus their effectiveness through the achieved drug concentration. The distribution of individual versions of polymorphisms may differ depending on the given population [21,22].

The variable prevalence of individual gene variants associated with drug metabolism pathways or their targets may affect the different effects of the applied treatment. Several scientific studies have found that belonging to a specific ethnic group may have a significant impact on the poorer response to treatment, even assuming that drug concentrations may reach different levels [21,22,23].

This leads us to questions that pharmacogenetics can answer. We should focus on analyzing a larger amount of data from different research centers, and therefore from different populations. We believe that the data provided by our team can be an important part of this analysis.

## 6. Conclusions

The response to paracetamol and ibuprofen in the treatment of HsPDA depends on many factors and mechanisms. Differences may result from the genetic variability of organisms. We are unaware of prior investigations evaluating the effect of these gene polymorphisms on pharmacologic ductal closure in HsPDA. Knowing and understanding it will give us a tool for more adequate treatment and will allow us to avoid adverse effects resulting from the use of drugs to which the response may be unsatisfactory.

Evidence indicates that distinct polymorphisms within the same genes may modulate diverse therapeutic outcomes. A limitation of our study is the homogeneous population of the study group, which consisted of newborns of the Caucasian population hospitalized in our center. Further advancement of research in this field is expected to facilitate the development of more effective treatment strategies.

## Figures and Tables

**Table 1:** Echocardiographic measures used to determine HsPDA.

Parameter	Criterion
Ductal diameter (maximum internal diameter from the pulmonary artery side)	⋝1.5 mm in infants < 27 weeks’ gestation ⋝2.5 1.5 mm in infants > 27 weeks’ gestation
PDA/LPA (Ratio of ductal diameter to left pulmonary artery diameter)	Small < 0.5 Moderate 0.5–1.0 Large > 1.0
La/ao (Left atrium to aortic root diameter ratio)	>1.8
Left ventricular enlargement	LV end-diastolic diameter (LVDd) according to Z-score
Descending aortic flow distal to the ductal insertion	Absence of diastolic flow or retrograde flow during diastole
End-diastoliv flow velocity in the left pulmonary artery	>0.2 m/s
Doppler flow in the anterior cerebral artery (ACA), renal arteries, and celiac trunk	Resistance index (RI) > 0.9

**Table 2:** The following single-nucleotide polymorphisms (SNPs) were analyzed.

Gene	Variant Number	Allels	Restrictions Enzymes
*PTGS1 (COX1)*	rs3842787	C>T	FauI
*PTGES2*	rs13283456	C>T	EcoNI
*PTGER4*	rs4613763	T>C	BccI
*PLA2G4A*	rs10798059	G>A	BanI
*PLA2G4C*	rs1549637	A>T	BstYI
*PLA2G6*	rs4375	T>C	AvrII
*PLA2G7*	rs1805017	C>T	BclI
*PLA2G7*	rs1051931	A>G	SatI

**Table 3:** Stratification of the Study Group by Demographic Parameters.

Characteristic												
	No PDA n = 42		*p*	PDA n = 36		*p*	HsPDA n = 21		*p*	PDA vs. HsPDA		*p*
Sex	Female	33	*p* = 0.0932	Female	12	*p* = 0.089	Female	7	*p* = 1.000	Female	12 vs. 7	*p* = 1.000
	Male	30		Male	24		Male	14		Male	24 vs. 14	
Gestational Age (week)	<28	21	*p* = 0.1350	<28	18	*p* = 0.111	<28	13	*p* = 0.176	<28	18 vs. 13	*p* = 0.552
	>28	42		>28	18		>28	8		>28	18 vs. 8	
Birth weight (grams)	<1000 g	17	*p* = 0.0498	<1000 g	17	*p* = 0.070	<1000 g	13	*p* = 0.080	<1000 g	17 vs. 13	*p* = 0.426
	>1000 g	46		>1000 g	19		>1000 g	8		>1000 g	19 vs. 8	
Prenatal steroid therapy	Yes	48	*p* = 0.4544	Yes	30	*p* = 0.524	Yes	18	*p* = 1.000	Yes	30 vs. 18	*p* = 1.000
	No	15		No	6		No	3		No	6 vs. 3	
Invasive ventilation	Yes	21	*p* = 0.0059	Yes	23	*p* = 0.0069	Yes	14	*p* = 0.9532	Yes	23 vs. 14	*p* = 0.552
	No	42		No	13		No	7		No	13 vs. 7	
Pharmacological ligation	Yes	0	*p* = 0.0001	Yes	21	*p* < 0.0001	Yes	21	*p* < 0.0001	Yes	21 vs. 21	*p* = 0.0017
	No	63		No	15		No	0		No	15 vs. 0	
Complications												
NEC	Yes	6	*p* = 0.0118	Yes	11	*p* = 0.012	Yes	5	*p* = 0.501	Yes	11 vs. 5	*p* = 0.895
	No	57		No	25		No	15		No	25 vs. 15	
IVH	Yes	21	*p* = 0.0879	Yes	19	*p* = 0.0629	Yes	14	*p* = 0.1017	Yes	19 vs. 14	*p* = 0.4554
	No	42		No	17		No	7		No	17 vs. 7	
BPD	Yes	27	*p* = 0.0064	Yes	26	*p* = 0.0075	Yes	15	*p* = 0.556	Yes	26 vs. 15	*p* = 1.0000
	No	36		No	10		No	6		No	10 vs. 6	
ROP	Yes	24	*p* = 0.0364	Yes	22	*p* = 0.048	Yes	13	*p* = 1.000	Yes	22 vs. 13	*p* = 1.000
	No	39		No	14		No	8		No	14 vs. 8	

**Table 4:** Association of Individual SNPs with Successful Pharmacologic Ductal Closure.

SNV	Model	Genotypes	Success N (%)	Failure N (%)	OR (95%CI)	*p*-Value	AIC
rs1236913	Codominant	CC	16 (88.9)	3 (100.0)	1.00	1.000	20.6
		CT	2 (11.1)	0 (0.0)	—		
rs13283456	Codominant	CC	18 (100.0)	3 (100.0)	—	—	—
rs4613763	Codominant	TT	13 (72.2)	0 (0.0)	1.00	0.042	14.6
		TC	5 (27.8)	3 (100.0)	—		
rs10798059	Codominant	GG	7 (38.9)	0 (0.0)	1.00	0.395	20.5
		GA	8 (44.4)	2 (66.7)	—		
		AA	3 (16.7)	1 (33.3)	—		
	Dominant	GG	7 (38.9)	0 (0.0)	1.00	0.521	18.5
		GA-AA	11 (61.1)	3 (100.0)	—		
	Recessive	GG-GA	15 (83.3)	2 (66.7)	1.00	0.521	20.8
		AA	3 (16.7)	1 (33.3)	2.50 (0.17–37.26)		
	Overdominant	GG-AA	10 (55.6)	1 (33.3)	1.00	0.473	20.7
		GA	8 (44.4)	2 (66.7)	2.50 (0.19–32.80)		
rs1549637	Codominant	TT	11 (61.1)	3 (100.0)	1.00	0.658	20.5
		TA	5 (27.8)	0 (0.0)	—		
		AA	2 (11.1)	0 (0.0)	—		
	Dominant	TT	11 (61.1)	3 (100.0)	1.00	0.521	18.5
		TA-AA	7 (38.9)	0 (0.0)	—		
	Recessive	TT-TA	16 (88.9)	3 (100.0)	1.00	1.000	20.6
		AA	2 (11.1)	0 (0.0)	—		
	Overdominant	TT-AA	13 (72.2)	3 (100.0)	1.00	0.549	19.4
		TA	5 (27.8)	0 (0.0)	—		
rs4375	Codominant	TT	3 (16.7)	1 (33.3)	1.00	1.000	22.0
		TC	12 (66.7)	2 (66.7)	0.50 (0.03–7.54)		
		CC	3 (16.7)	0 (0.0)	—		
	Dominant	TT	3 (16.7)	1 (33.3)	1.00	0.521	20.8
		TC-CC	15 (83.3)	2 (66.7)	0.40 (0.03–5.96)		
	Recessive	TT-TC	15 (83.3)	3 (100.0)	1.00	1.000	20.2
		CC	3 (16.7)	0 (0.0)	—		
	Overdominant	TT-CC	6 (33.3)	1 (33.3)	1.00	1.000	21.2
		TC	12 (66.7)	2 (66.7)	1.00 (0.07–13.37)		
rs1805017	Codominant	CC	11 (61.1)	0 (0.0)	1.00	0.090	16.2
		CT	7 (38.9)	3 (100.0)	—		
rs1051931	Codominant	GG	9 (50.0)	1 (33.3)	1.00	1.000	22.5
		GA	8 (44.4)	2 (66.7)	2.25 (0.17–29.77)		
		AA	1 (5.6)	0 (0.0)	—		
	Dominant	GG	9 (50.0)	1 (33.3)	1.00	0.589	20.9
		GA-AA	9 (50.0)	2 (66.7)	2.00 (0.15–26.18)		
	Recessive	GG-GA	17 (94.4)	3 (100.0)	1.00	1.000	20.9
		AA	1 (5.6)	0 (0.0)	—		
	Overdominant	GG-AA	10 (55.6)	1 (33.3)	1.00	0.473	20.7
		GA	8 (44.4)	2 (66.7)	2.50 (0.19–32.80)		

**Table 5:** Drug-specific effects were observed, with evidence of SNP-by-treatment interaction for paracetamol versus ibuprofen in relation to pharmacologic closure.

SNV	Model	Genotypes	Ibuprofen N (%)	Paracetamol N (%)	OR (95%CI)	*p*-Value	AIC
rs1236913	Codominant	CC	2 (66.7)	13 (92.9)	1.00	0.256	18.6
		CT	1 (33.3)	1 (7.1)	0.15 (0.01–3.58)		
rs13283456	Codominant	CC	3 (100.0)	14 (100.0)	—	—	—
rs4613763	Codominant	TT	3 (100.0)	8 (57.1)	1.00	0.515	16.9
		TC	0 (0.0)	6 (42.9)	—		
rs10798059	Codominant	GG	2 (66.7)	4 (28.6)	1.00	0.290	18.1
		GA	0 (0.0)	7 (50.0)	—		
		AA	1 (33.3)	3 (21.4)	1.50 (0.09–25.39)		
	Dominant	GG	2 (66.7)	4 (28.6)	1.00	0.220	18.3
		GA-AA	1 (33.3)	10 (71.4)	5.00 (0.35–71.90)		
	Recessive	GG-GA	2 (66.7)	11 (78.6)	1.00	0.669	19.7
		AA	1 (33.3)	3 (21.4)	0.55 (0.04–8.27)		
	Overdominant	GG-AA	3 (100.0)	7 (50.0)	1.00	0.228	16.2
		GA	0 (0.0)	7 (50.0)	—		
rs1549637	Codominant	TT	3 (100.0)	7 (50.0)	1.00	0.669	18.2
		TA	0 (0.0)	5 (35.7)	—		
		AA	0 (0.0)	2 (14.3)	—		
	Dominant	TT	3 (100.0)	7 (50.0)	1.00	0.228	16.2
		TA-AA	0 (0.0)	7 (50.0)	—		
	Recessive	TT-TA	3 (100.0)	12 (85.7)	1.00	1.000	19.0
		AA	0 (0.0)	2 (14.3)	—		
	Overdominant	TT-AA	3 (100.0)	9 (64.3)	1.00	0.515	17.5
		TA	0 (0.0)	5 (35.7)	—		
rs4375	Codominant	TT	1 (33.3)	3 (21.4)	1.00	1.000	20.9
		TC	2 (66.7)	9 (64.3)	1.50 (0.10–23.07)		
		CC	0 (0.0)	2 (14.3)	—		
	Dominant	TT	1 (33.3)	3 (21.4)	1.00	0.669	19.7
		TC-CC	2 (66.7)	11 (78.6)	1.83 (0.12–27.80)		
	Recessive	TT-TC	3 (100.0)	12 (85.7)	1.00	1.000	19.0
		CC	0 (0.0)	2 (14.3)	—		
	Overdominant	TT-CC	1 (33.3)	5 (35.7)	1.00	0.937	19.8
		TC	2 (66.7)	9 (64.3)	0.90 (0.06–12.58)		
rs1805017	Codominant	CC	0 (0.0)	8 (57.1)	1.00	0.206	15.5
		CT	3 (100.0)	6 (42.9)	—		
rs1051931	Codominant	GG	2 (66.7)	6 (42.9)	1.00	0.451	19.3
		GA	1 (33.3)	8 (57.1)	2.67 (0.19–36.75)		

## Data Availability

The data presented in this study are available on request from the corresponding author due to data privacy regulations, the datasets generated and/or analyzed during the current study are not publicly accessible.
